# Acute ST-elevation myocardial infarction (STEMI) in a young man with unknown multiple and mixed Valvular heart diseases: case report

**DOI:** 10.1093/omcr/omae074

**Published:** 2024-07-21

**Authors:** Youssef Lahmouz, Frederick Nana, Soumia Faid, Driss Britel, Nadia Loudiyi, Hicham Faliouni, Najat Mouine, Zouhair Lakhal, Aatif Benyass

**Affiliations:** Clinical Cardiology Department, Cardiology Center, Mohammed V Military Instruction Hospital of Rabat, Mohammed V University, Morocco; Intensive Cardiac Care, Cardiology Center, Mohammed V Military Instruction Hospital of Rabat, Mohammed V University, Morocco; Intensive Cardiac Care, Cardiology Center, Mohammed V Military Instruction Hospital of Rabat, Mohammed V University, Morocco; Intensive Cardiac Care, Cardiology Center, Mohammed V Military Instruction Hospital of Rabat, Mohammed V University, Morocco; Clinical Cardiology Department, Cardiology Center, Mohammed V Military Instruction Hospital of Rabat, Mohammed V University, Morocco; Intensive Cardiac Care, Cardiology Center, Mohammed V Military Instruction Hospital of Rabat, Mohammed V University, Morocco; Clinical Cardiology Department, Cardiology Center, Mohammed V Military Instruction Hospital of Rabat, Mohammed V University, Morocco; Intensive Cardiac Care, Cardiology Center, Mohammed V Military Instruction Hospital of Rabat, Mohammed V University, Morocco; Head of Cardiology Center, Mohammed V Military Instruction Hospital of Rabat, Mohammed V University, Morocco

**Keywords:** cardiology and cardiovascular systems, critical care medicine, pharmacology and pharmacy

## Abstract

Although the incidence of systemic thromboembolism in valvular heart disease has been reported to be as high as 10% to 35%, embolization to the coronary arteries is uncommon. We present a case of a patient with acute myocardial infarction caused by coronary thromboemboli associated with combined valvular heart disease and atrial fibrillation. The thromboemboli were documented in the left descending artery. Coronary interventions including thromboaspiration and percutaneous coronary balloon angioplasty were attempted.

## Introduction

In the Moroccan context of endemic rheumatic fever, mitral stenosis (MS) is the most common valvulopathy. MS is primarily known for its upstream consequences, among which atrial rhythm disorders, such as atrial fibrillation (AF), and systemic embolisms are prominent. The latter are common at the cerebral level and very rare at the coronary level. We report the case of a patient in whom Multiple and Mixed Valvular Heart Diseases was revealed by a coronary embolism, leading to a myocardial infarction (MI).

## Case report

A 42-year-old man presented to the emergency department with retrosternal crushing pain of sudden onset 1H before the admission.

He had no modifiable cardiovascular risk factors and he suffered from progressive exertional and nocturnal dyspnea for the previous year. Vital signs revealed an irregular pulse at a rate of 100 beats per minute, a blood pressure of 140/70 mm Hg, respiratory rate of 14 breaths per minute, and a temperature of 36.7°C.

Precordial examination revealed a tapping apical impulse, loud first heart sound and pulmonary component of second heart sound, opening snap, and a long mid-diastolic rumble over the apex. Other systems were normal.

**Figure 1:**
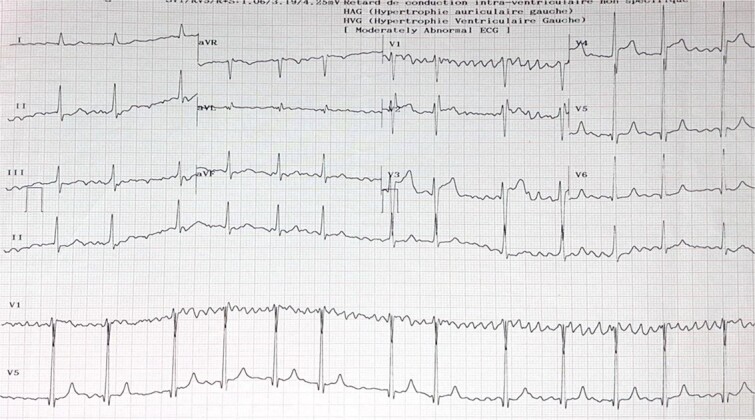
EKG showed atrial fibrillation and about 2 mm ST segment elevation in leads V3, V4.

**Figure 2:**
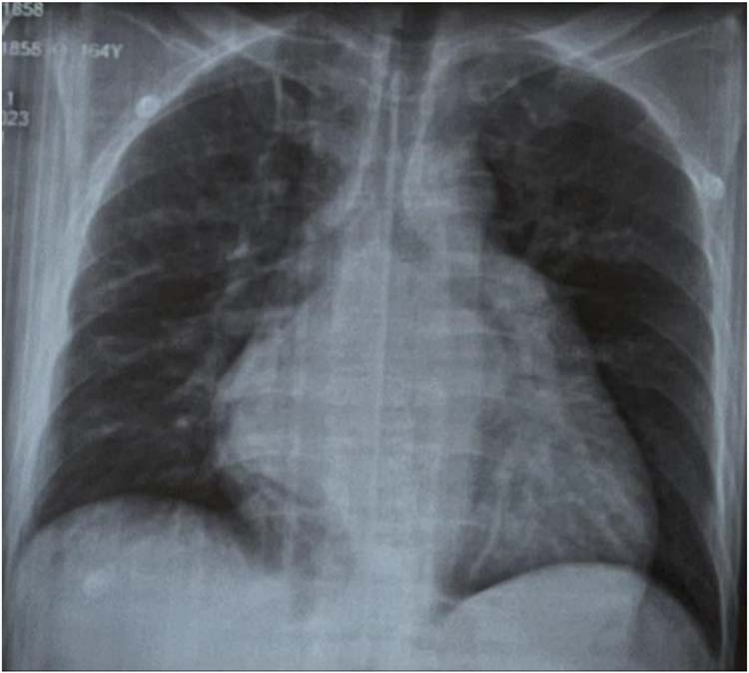
Chest radiography revealed cardiomegaly with radiological signs of pulmonary arterial and venous hypertension and biatrial enlargement.

An electrocardiogram showed atrial flutter with variable conduction and about 2 mm ST segment elevation in leads V3, V4 (Fig. 1). A chest radiography showed cardiomegaly with radiological signs of pulmonary arterial and venous hypertension and biatrial enlargement (Fig. 2).

Patient was loaded with acetyl salicylic acid 300 mg and clopidogrel 600 mg as antithrombotic agents and enoxaparin 0.3 mg intravenous for anticoagulation.

Coronary angiography showed thrombus in the mid left anterior descending artery. There were no atherosclerotic plaques or stenotic lesions in either the left or the right coronary systems (Fig. 6); Intravascular Ultrasound and Optical Coherence Tomography were not used to rule out coronary atherosclerosis. Thromboasipiration failed to recanalize this occlusion. Subsequent balloon angioplasty of the left left anterior descending artery was performed with success (Fig. 7). Anti glycoprotein IIb-IIIa in combination with unfractionated heparin was administrated for 24 h. The diagnosis of coronary artery embolism (CE) was made, based on the proposed national cerebral and cardiovascular center (NCVC) criteria for the clinical diagnosis of coronary artery embolism (CE).

**Figure 3:**
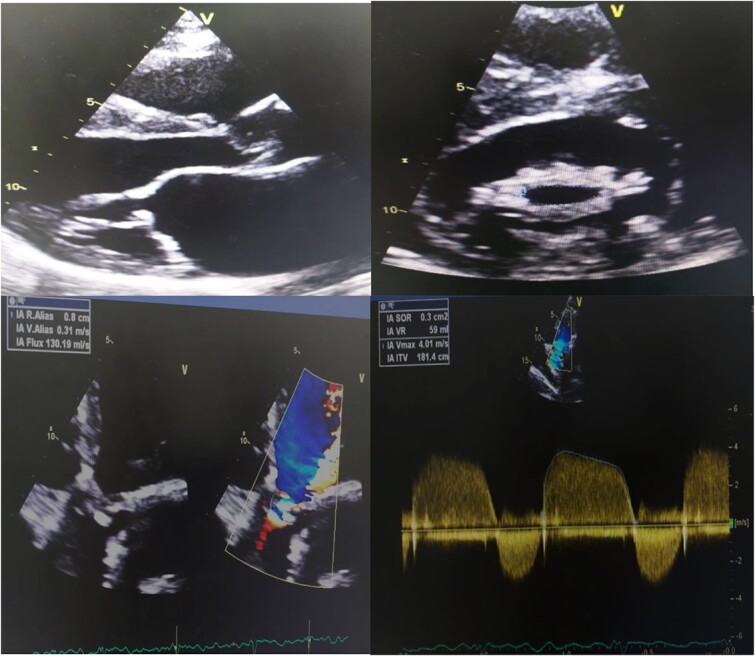
A transthoracic echocardiography showed severe mitral stenosis with severe aortic regurgitation and biatrial enlargement. It also reveals segmental ventricular wall motion abnormalities: akinesis of the apex and basal and medial anterolateral wall with preserved fraction ejection at 50%.

Transthoracic echocardiography day after showed thickened and fused mitral valve leaflets with annular calcification, chordal shortening restricting the leaflet motion. The mean diastolic gradient across the mitral valve was estimated to 8 mmHg and the valve area calculated to be 1.4 cm2. There was a biatrial dilatation and the calculated pulmonary artery systolic pressure was 55 mm Hg. The aortic valve was calcificated with restriction of the cusps with severe aortic regurgitation. It also revealed segmental ventricular wall motion abnormalities: akinesis of the apex and basal and medial anterolateral wall with preserved fraction ejection at 50% (Fig. 3).

**Figure 4:**
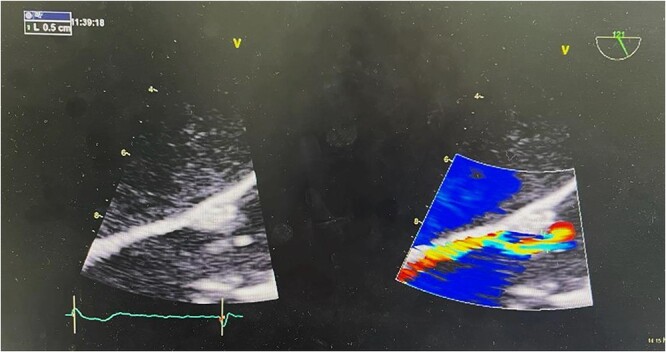
Transesophageal echocardiography showed severe aortic regurgitation.

**Figure 5:**
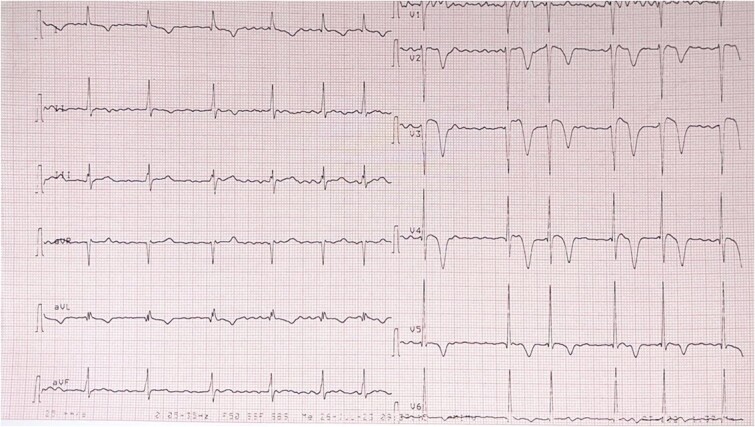
Post-procedural EKG showed atrial fibrillation and T-wave inversion in the anterior leads.

A subsequent transesophageal exam confirmed these findings (Fig. 4). Post-procedural EKG showed normal sinus rhythm and T-wave inversion in the inferior leads (Fig. 5).

**Figure 6:**
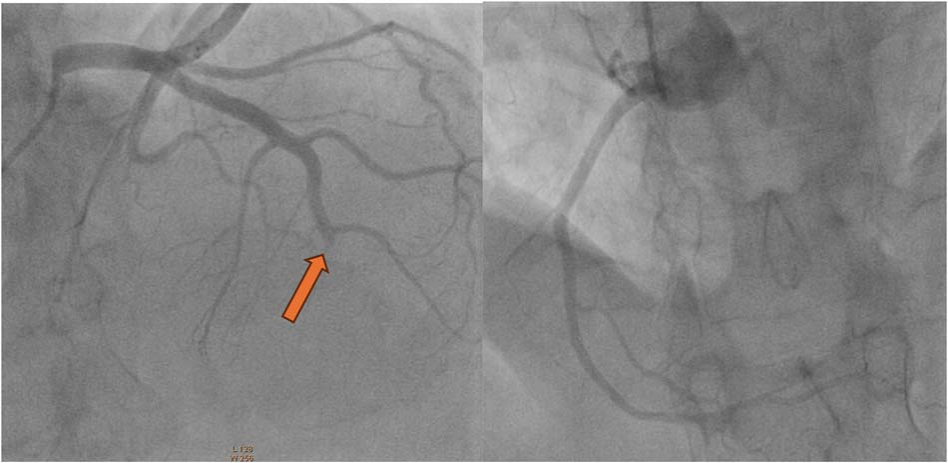
Coronary angiography showed thrombus in the mid left anterior descending artery (Yellow arrow) with no atherosclerotic plaques or stenotic lesions in either the left or the right coronary systems.

**Figure 7:**
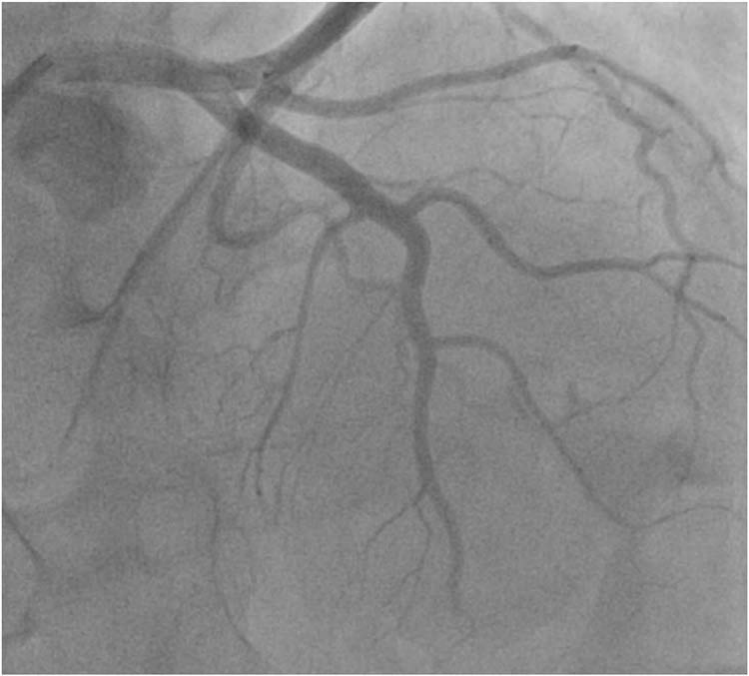
Coronagraphy of control showed the dissolution of the thrombus treated with thromboaspiration and balloon inflation with anti glycoprotein IIb-IIIa in combination with unfractionated heparin.

Biological workup revealed a positive troponin at 539 ng/ml and concentration peaked at 20 256 ng/ml.

The patient was treated with acénocoumarol, aspirin, clopidogrel, bisoprolol and spironolactone. With the treatment, the patient experienced symptomatic relief and hemodynamic stability, and he was referred for mitral and aortic valve replacement.

## Discussion

Systemic embolism can have its origin in thrombosis, often localized in the left atrial appendage, sometimes lining a wall of the left atrium, and exceptionally massive [1].

The incidence of systemic embolism is reported to be between 9.6% to 18% in surgical series [1, 2], and it rises to 41% in autopsy series [3]. The main predisposing factor is the prior occurrence of atrial fibrillation (AF), which is observed in 90% of cases at the time of embolism.

Coronary embolism-induced myocardial infarction (MI) is a rare condition. The majority of emboli (75%) affect the left coronary network [1, 2]. This is likely due to its larger diameter and the less acute angle of implantation compared to the right coronary artery [3].

The etiology of coronary embolism is often associated with valvular heart disease, prosthetic valve usage, infective endocarditis, dilated cardiomyopathy, and arrhythmias [4].

Paradoxical embolism can also occur as a result of venous thrombosis in atrial septal defects [5].

Intracardiac thrombus causing coronary embolism can originate from the atria or the ventricles due to stagnant blood flow. Intraventricular thrombus is typically caused by ventricular aneurysm, severe left ventricular systolic dysfunction, or kinetic disorders [6]. However, no evidence of all-cause ventricular stasis was found in this particular case.

Predisposing factors for thrombus formation in this case included intra atrial enlargement, mitral valve disease, and atrial fibrillation, which can lead to reduced blood flow in the left atrium. The highest incidence of left atrial thrombus is associated with rheumatic mitral stenosis and atrial fibrillation [6].

The criteria usually required are:

- Coronary angiographic appearance
- Identification of the origin of the thrombus.
- The normality of the endocoronary intima.

Or the third criterion, intracoronary ultrasound was not performed, but the rest of the coronary network did not show any atheromatous lesions. This alone is already an accepted argument in favor of the diagnosis [3].

Percutaneous transluminal coronary angioplasty with stent placement can be performed successfully as demonstrated by Sial and al. in patients with embolic myocardial infarction [7]. Another alternative to percutaneous coronary angioplasty is performing the procedure without stenting. Some authors have described aspiration thrombectomy as an effective management approach, although it did not yield the desired outcome in our case [8].

Using anticoagulants and high-dose glycoprotein IIb/IIIa inhibitors for thrombus dissolution, particularly when the risk of bleeding is low, could be useful. In this specific case, we achieved successful recanalization of the left anterior descending artery (LAD) by inflating a balloon following 48 h of intravenous anticoagulation with the glycoprotein IIb/IIIa antagonist tirofiban, in combination with heparin (HNF). This approach resulted in satisfactory control without any bleeding complications. It’s worth noting that we did not employ fibrinolytics such as streptokinase or urokinase in this case.

## Conclusion

Coronary embolism-induced myocardial infarction (MI) is a rare condition. Mitral stenosis (MS) is the most common valvulopathy etiology associated with coronary embolism. Early surgical treatment would be the aim to avoid this complication.
